# A scoping review of cultural issues concerning institutional quarantine and isolation during major multi‐country outbreaks in Africa: 2000–2023

**DOI:** 10.1002/hsr2.70114

**Published:** 2024-09-29

**Authors:** Ebunoluwa Oduwole, Jimoh Amzat, Olusola Aluko‐Arowolo, Rotimi Afolabi, Isaac Akinkunmi Adedeji, Saheed Akinmayowa Lawal, Ige Angela Temisan, Ayoyinka Oludiran, Kafayat Aminu, Afeez Abolarinwa Salami, Kehinde Kazeem Kanmodi

**Affiliations:** ^1^ Department of Philosophy Olabisi Onabanjo University Ago‐Iwoye Nigeria; ^2^ Department of Sociology Usmanu Danfodiyo University Sokoto Nigeria; ^3^ Department of Sociology Olabisi Onabanjo University Ago‐Iwoye Nigeria; ^4^ Department of Epidemiology and Medical Statistics, College of Medicine University of Ibadan Ibadan Nigeria; ^5^ Department of Sociology Hallmark University Ijebu‐Itele Nigeria; ^6^ Department of Public Health Babcock University Ilishan‐Remo Nigeria; ^7^ Department of Science and Technology Education University of Ibadan Ibadan Nigeria; ^8^ Center for Child and Adolescent Mental Health University College Hospital Ibadan Nigeria; ^9^ Department of Oral and Maxillofacial Surgery University College Hospital Ibadan Nigeria; ^10^ Department of Public Health Dentistry Manipal Academy of Higher Education Manipal India; ^11^ Cephas Health Research Initiative Inc Ibadan Nigeria; ^12^ School of Dentistry University of Rwanda Kigali Rwanda; ^13^ Faculty of Dentistry University of Puthisastra Phnom Penh Cambodia

**Keywords:** Africa, cultural issues, outbreaks, pandemic, quarantine and isolation, review

## Abstract

**Background and Aims:**

Infectious disease outbreaks pose significant challenges in Africa due to its ecology, socioeconomic conditions, and weak health systems. Implementing institutional quarantine and isolation (Q&I) measures is crucial for managing major outbreaks. However, the cultural context often determines the success of these measures. This scoping review aims to examine existing evidence on the cultural aspects of institutional Q&I in Africa over the past two decades, focusing on the COVID‐19 pandemic and other major multi‐country disease outbreaks.

**Methods:**

This scoping review's protocol was registered with the Open Science Framework registry. Nine research databases were systematically searched to retrieve all relevant literature, followed by deduplication and a two‐stage screening process using the Rayyan web application. The inclusion of any literature into this review was based on a set of eligibility criteria. Also, manual searching of the reference lists of included literature was done to retrieve any other eligible literature. From the included literature, data were charted, collected, and summarized.

**Findings:**

Out of the 787 articles retrieved from the database searches, this review found only one to be eligible for inclusion. Also, no other eligible article was obtained after manual searching of the reference list for this article. The reviewed article presented empirical findings on the impact of COVID‐19 Q&I protocols on traditional burial rites in Ghana. Many Ghanaian families were against their country's protocol because they felt it was insensitive to their traditional values and burial practices. Also, the way the protocol was implemented made some Ghanaian families feel that foreign burial practices were imposed on them, which bred feelings of cultural exclusion and neglect (by the government) among Ghanaians.

**Conclusion:**

Research evidence on the cultural implications of Q&I in Africa is very scanty. More research is needed on this topic of public health interest.

## 1 INTRODUCTION

The emergence and rapid spread of infectious diseases pose significant challenges to global public health, necessitating coordinated responses to mitigate the impact of outbreaks.^1^, ^2^, ^3^ It has been observed that the ecology, socioeconomic condition and weak health systems make Africa an area favourable to the occurrence of various diseases and disease outbreaks.^3^ In the African context, where the burden of infectious diseases is significant, institutional quarantine and isolation (Q&I) measures become critical for containing and managing major multi‐country outbreaks.^4^, ^5^ Notable outbreaks such as Ebola, Lassa fever, monkeypox (mpox), and the global coronavirus of 2019 (COVID‐19) pandemic have marked the landscape of infectious diseases in Africa.^6^ In addition to causing public panic, these multi‐country infectious diseases disrupted various activities, including human mobility, and necessitated countermeasures such as screening and Q&I.^7^ Unfortunately, the infrastructure available to confront such outbreaks is often minimal, thereby overwhelming the health system.^8^ There remain critical challenges to implementing Q&I in a country with different and unique social contexts.^9^, ^10^

In response to these threats, governments and health authorities across the continent have often turned to institutional Q&I strategies to curb the transmission of infectious agents.^4^, ^5^ However, the cultural context in which we implement such measures intricately determines their success and effectiveness.^11^, ^12^ There has always been resistance to Q&I due to public misunderstanding and attendant social constraints, especially in the form of movement restrictions and social separation for several days.^7^, ^10^

Cultural factors play a pivotal role in shaping how communities and individuals respond to and experience Q&I.^13^ Cultural beliefs, practices, and norms influence perceptions of health and illness, attitudes towards authority, and the willingness to comply with public health directives.^14^ Additionally, considerations of equity, human rights, and the potential for stigmatization further underscore the need for a nuanced understanding of the cultural dimensions of institutional Q&I in Africa.^15^ This scoping review seeks to analyze the existing literature on the cultural aspects of institutional quarantine and isolation across African countries over the past two decades. By adopting a broad approach, the review aims to capture the diversity of experiences and responses within different cultural contexts. Through a comprehensive examination of peer‐reviewed articles, we aim to identify gaps and key themes that have emerged in the discourse on cultural issues concerning Q&I implementation in Africa.

The temporal scope of 2000 to 2023 is deliberately selected to encompass a period marked by both historical infectious disease challenges and the more recent global health crisis triggered by the COVID‐19 pandemic.^6^ Understanding the evolution of cultural perspectives and responses during this timeframe could provide valuable insights into the adaptability and resilience of African societies in the face of emerging health threats. The scoping review methodology allows for a systematic exploration of the literature, facilitating a comprehensive overview of the existing knowledge landscape. By adopting a multidisciplinary approach, this review aims to bring together insights from public health, anthropology, sociology, and related fields to provide a holistic understanding of the cultural dimensions of institutional Q&I in Africa. Specifically, this review examines existing empirical evidence and gaps on cultural issues concerning institutional quarantine and isolation during viral pandemics (and near pandemics) in Africa. This scoping review seeks to contribute to the existing body of knowledge, offering a nuanced perspective that can inform future public health interventions, policies, and strategies tailored to the unique cultural contexts of the continent.

## 2 METHODS

### 2.1 Research design & protocol registration

This scoping review was conducted by using a synthesis of the five stages of methodological evaluation of scoping reviews.^16^ Also, the title and protocol of this scoping review have been registered with the Open Science Framework registry.

### 2.2 Research question

The research question this scoping review seeks to answer is “What empirical evidence and gaps exist on the cultural issues concerning institutional quarantine and isolation during viral pandemics (and near pandemics) in Africa?”

### 2.3 Identification of relevant literature

On 24 June 2023, an online systematic search of nine research databases (SCOPUS, PubMed, Allied and Complementary Medicine Database (AMED), CINAHL Ultimate, APA PsycArticles, APA PsycInfo, Dentistry and Oral Sciences Source, SPORTDiscus with Full Text, and Psychology and Behavioral Sciences Collection) was conducted to identify and retrieve articles published between 2000 and 2023 using a combination of relevant search terms, Boolean operators (“AND” and “OR”), and truncations (“*” or “#”) (Tables S1 to S3; Supplementary file). The search terms (full/truncated word) used for the literature search include ‘quarantine’, ‘isolat*’, ‘separat*’, ‘seclu*’, ‘deten*’, ‘Ebola’, ‘COVID*’, ‘corona*’, ‘sars‐cov‐2’, ‘Lassa’, ‘pandemic’, ‘outbreak’, ‘widespread’, ‘Algeria’, ‘Angola’, ‘Benin’, ‘Botswana’, ‘Burkina Faso’, ‘Burundi’, ‘Cape Verde’, ‘Cabo Verde’, ‘Cameroon’, ‘Central African Republic’, ‘Chad’, ‘Comoros’, ‘Congo’, ‘Cote d'Ivoire’, ‘Ivory Coast’, ‘Djibouti’, ‘Democratic Republic of Congo’, ‘Egypt’, ‘Equatorial Guinea’, ‘Eritrea’, ‘Eswatini’, ‘Ethiopia’, ‘Gabon’, ‘Gambia’, ‘Ghana’, ‘Guinea’, ‘Guinea Bissau’, ‘Kenya’, ‘Lesotho’, ‘Liberia’, ‘Libya’, ‘Madagascar’, ‘Malawi’, ‘Mali’, ‘Mauritania’, ‘Mauritius’, ‘Morocco’, ‘Mozambique’, ‘Namibia’, ‘Niger’, ‘Nigeria’, ‘Rwanda’, ‘Sao Tome and Principe’, ‘Senegal’, ‘Seychelles’, ‘Sierra Leone’, ‘Somalia’, ‘South Africa’, ‘South Sudan’, ‘Sudan’, ‘Tanzania’, ‘Togo’, ‘Tunisia’, ‘Uganda’, ‘Zambia’, ‘Zimbabwe’, ‘Reunion’, ‘Saint Helena’, ‘Western Sahara’, and ‘Mayotte’.

### 2.4 Selection of literature

All the retrieved articles were imported into the Rayyan web application for the deduplication of all duplicate records.^17^ The deduplicated articles were subjected to a two‐stage screening process by two independent reviewers who were guided by a set of specific eligibility criteria. The first stage of screening involved title and abstract screening of the deduplicated literature to identify those literature that are relevant at prima facie. The nonrelevant literature identified at the first‐stage screening were excluded while the remaining ones were subjected to a second‐stage screening. In the second stage of screening, a full‐text evaluation of the residual literature was done. Following the full‐text evaluation, only those articles that met the inclusion criteria were included in the review. Also, during the two‐stage screening process, all conflicts were resolved through thorough discussion between the two reviewers. Also, forward and backward chain‐referencing was done. The reference lists of the included literature were manually searched to identify additional literature for inclusion in the review.

In this scoping review, only those literature that met the following criteria were included:

- Peer‐reviewed journal articles
- Articles published in English
- Articles reporting empirical findings on the cultural issues concerning institutional quarantine and isolation during COVID‐19, Ebola virus disease, and Lassa fever outbreaks in Africa
- Articles published between the year 2000 and 2023 (as at 24 June 2023)
- Articles with accessible full texts

However, the exclusion criteria include:

- Grey literature (e.g., books, book chapters, magazines, etc.)
- Unmatched publication types such as peer‐reviewed literature that were not original research journal articles (e.g., systematic reviews, letters, opinions, comments, editorials, etc.)
- Literature that was published in other languages aside from English
- Literature that reported empirical findings on the cultural issues concerning institutional quarantine and isolation during non‐COVID‐19, non‐Ebola virus disease, and non‐Lassa fever outbreaks in Africa
- Literature that did not report empirical findings on the cultural issues concerning institutional quarantine and isolation during COVID‐19, Ebola virus disease, and Lassa fever outbreaks in Africa
- Literature published before the year 2000 and after the date of the literature search (24 June 2023).
- Unmatched study types, objectives, or study outcomes.

### 2.5 Data charting, collation, and summarisation

From the included articles, data were extracted using a bespoke data extraction sheet. We adopted the Preferred Reporting Items for Systematic Reviews and Meta‐Analyses extension for Scoping Reviews (PRISMA‐ScR). The data extracted include names of authors, year of publication, authors' names, country/location of the study, sample size, study population/sample characteristics, study design, study instruments, findings, and conclusions. The extracted data were collated, summarized, and presented in texts and tables.

## 3 RESULTS

**Figure 1:**
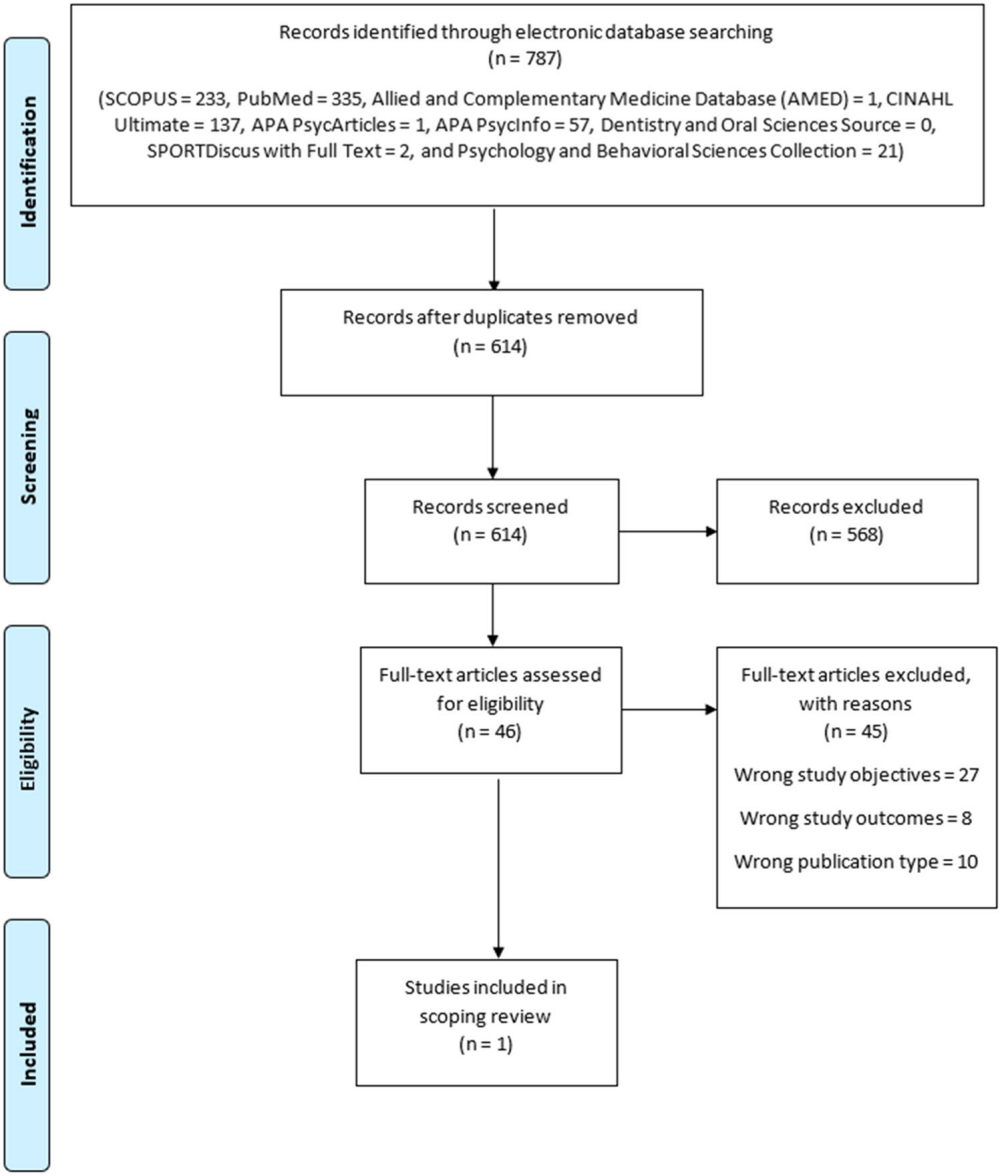
PRISMA Flow chart of literature search and screening process.

A total of 787 literature were obtained from the electronic database search. After the removal of duplicates, 614 single‐entry literature were remaining and screened for eligibility. After a two‐stage screening, only 1 article/study was included in the review (Table S4; Supplementary file). Also, from the manual search of the reference lists of the included article, no eligible article was identified. Finally, the data from only 1 article were charted, collated, and summarized in this scoping review (Figure 1).

### 3.1 Characteristics of the included article

Table 1 shows the summary of the included article.^18^ The article was on a qualitative study which explored the experiences of the participants while they were navigating/enforcing COVID‐19‐related death and burial protocols as well as the impact of the enforcement of COVID‐19‐related death and burial protocol on traditional burial and funeral rites in Cape Coast Metropolis, Ghana. The study investigated health workers and COVID‐19‐related bereaved family members.

**Table 1:** Summary of the Included article.

Author (year)	Study type (study design)	Country (location)	Setting	Sample characteristics	Data collection technique	Virus	Institution of Focus	Study Objectives	Relevant Conclusion
Takyiakwaa et al. (2023)^18^	Qualitative study (ethnographic design)	Ghana (Cape Coast Metropolis)	Community and hospital settings	19 participants (6 – public health workers enforcing adherence to COVID‐19‐related deaths and burial protocols; 13 – COVID‐19‐related bereaved family members; 17 – males; 2 – females)	Key informant interviews	COVID‐19	Government	To explore the: 1) experiences of the participants while they were navigating/enforcing COVID‐19‐related death and burial protocols 2) impact of the enforcement of COVID‐19‐related death and burial protocol on traditional burial and funeral rites in Ghana	The implementation of COVID‐19‐related death and burial protocols was compromised by the lack of sensitivity to sociocultural practices

### 3.2 Findings

The empirical findings summarized from the included study were grouped into five themes, which are presented below. These findings were based only on the Ghanaian cultural landscape, as no other relevant African study was available for the data synthesis.

#### 3.2.1 Cultural norms in the care of the dead

In Ghana, the care of the dead is a deeply rooted cultural issue; it is seen as a compulsory act, which is done to honour a dead person. The act often involved washing and dressing of the dead, wake keeping, pouring of libation, and some specific burial rites at the grave site.^18^ Furthermore, the observance of this practice was believed to bring blessings to the living relatives of the dead while its nonobservance is believed to bring supernatural punishments.^18^

#### 3.2.2 Attitudes and perceptions toward institutionally enforced burial practices

During the COVID‐19 outbreak in Ghana, the government enforced an isolated burial protocol for people who died of the disease.^18^ However, many Ghanaian family members were against the protocol because they felt it was insensitive to their cultural values and practices; hence, the protocol was highly resisted by many Ghanaian families.^18^ Furthermore, some Ghanaian family members perceived this enforcement as a neglect of the local traditions of the people by the government, and as an act of disrespect to Ghanaian families.^18^ This experience made some family members felt that foreign burial processes were being imposed on them.^18^

Many Ghanaian families also identified that they lacked information on how family members of persons who died of COVID‐19 can prepare and participate in the institutionally implemented burial processes.^18^ This further precipitated the feeling of exclusion and cultural neglect by the government.^18^

#### 3.2.3 Cultural implications of “uncultural” burial practices enforced by institutions

The widely perceived “uncultural” burial practices enforced by the Ghanaian government have cultural implications. Some participants reported that being deprived of the opportunity to bury their dead made them feel that their deceased were treated like a worthless entity by the government.^18^

## 4 DISCUSSION

In an African context, there has not been extensive research on the cultural implications of Q&I. Hence, this scoping review only included one eligible article. However, the findings obtained in this scoping review are noteworthy and have implications for public health research, policy, and practice. The findings are mainly about the enforcement of COVID‐19‐related death and burial protocols as well as the impact of the enforcement of COVID‐19‐related death and burial protocols on traditional burial and funeral rites in Ghana. Beyond the burial practices, it is important to examine the broad cultural issues concerning Q&I, including social distancing, cultural perceptions of public health emergencies, stigmatisation, traditional grieving practices, and customs of mourning and bereavement.

As reflected in the core finding of the reviewed article, funeral practices are significant cultural rites (18). Such rites, deeply rooted in cultural and religious practices, undergo significant adaptations during times of quarantine and isolation.^19^ The COVID‐19 pandemic, in particular, has compelled societies worldwide to re‐evaluate traditional mourning processes and rituals to address public health concerns while respecting the emotional needs of grieving families.^20^ One of the primary challenges during a pandemic is the restriction on large gatherings, including funeral ceremonies.^18^, ^21^, ^22^ Many cultures traditionally emphasise the importance of communal support during times of loss, where friends and family come together to mourn, share memories, and offer condolences. However, public health emergency measures necessitate a limited number of attendees, often leaving families to grapple with the difficult decision of selecting a small group to attend the funeral. This is a critical cultural and emotional issue, as the restriction may intensify the grieving process because individuals are unable to enjoy the collective strength of a larger community.

The interruptions to traditional grieving practices necessitate virtual funerals as a response to the restrictions on physical gatherings.^23^ Livestreamed funeral services enable friends and family to participate remotely, providing a sense of connection during a period when physical presence is limited.^24^ While this digital adaptation has proven valuable in maintaining a semblance of community support, it also underscores the technological disparities that exist, as not everyone may have access to the necessary resources for virtual participation. Funeral ceremonies during quarantine and seclusion must strike a careful balance between public health concerns and cultural and emotional demands. Communities and individuals all across the world are forced to adapt, devising new methods to honour the deceased while negotiating the hurdles given by limits on traditional traditions.^25^ The ability of human cultures to alter funeral ceremonies during times of crisis demonstrates the profound relevance of these rituals in bringing consolation, meaning, and a feeling of closure to people who have experienced loss.

Cultural and religious practices related to body preparation and viewing also face modifications during a public health emergency.^26^ In African cultures, there is a strong emphasis on viewing the deceased as a final farewell and as a way to acknowledge the reality of death. However, safety concerns surrounding the transmission of infectious diseases may limit or even prohibit these practices. Families may need to adapt to alternative methods of saying goodbye, such as through virtual viewings or small, controlled gatherings.^24^ Quarantine measures may also impact the process of burial or cremation. Safety protocols may require adjustments to certain cultures' specific rituals related to the handling and interment of the deceased.^26^ Additionally, limitations on the number of attendees at burial sites may lead to a re‐evaluation of burial traditions that involve a larger community presence.

Quarantine measures also affect the customs of mourning and bereavement. Traditionally, individuals come together to offer condolences, share meals, and provide emotional support to grieving families. Quarantine‐imposed isolation disrupts these acts of compassion, forcing mourners to navigate their grief in a more solitary manner. Grief is a deeply personal experience, and the restrictions on social interactions may intensify feelings of loneliness and isolation. Cultures that place a strong emphasis on communal mourning rituals may find it challenging to reconcile the need for physical distance with the cultural importance of shared grieving experiences.

One of the broad cultural issues includes the lack of social interaction and limited social support that epitomize institutional Q&I as superficially a disguise for temporary social exclusion. Similarly, social or physical distancing is a form of social avoidance that negates the principles of communal living and social cohesion, which are valued in most cultures. Research has shown that social isolation or exclusion can intensify pre‐existing illness symptoms and potentially trigger a new diagnosis.^27^, ^28^

The implementation of Q&I measures during public health crises brings forth a multitude of cultural issues that significantly influence individuals and communities.^29^ The intersection of cultural norms, values, and practices with these public health strategies can shape people's responses, compliance, and overall well‐being during times of public health emergencies.^30^ Impliedly, cultural norms and religious practices within communities often affect safety measures during an outbreak or pandemic.^31^ In general, cultural practices are significant determinants of human well‐being, and health and illness behavior in particular.^32^ Culture may influence exposure to, early detection of, and treatment of infectious diseases.^33^ Specifically, cultural greetings, such as handshakes or kisses on the cheek, may help to spread viruses and bacteria.^34^

Public health interventions should consider cultural beliefs and assumptions.^34^ Cultural perceptions or local understandings of health and illness vary widely across societies and cultures.^35^ In some cultures, the emphasis on collective well‐being may lead to a more communal acceptance of Q&I measures as altruistic measures for protecting others. People may view these restrictions as a necessary sacrifice for the greater good, aligning with the cultural values of solidarity and responsibility towards the community.^36^ Conversely, many laypeople and scientists contend that mass quarantines are necessary for the public.^37^ However, in other cultures that prioritize individual autonomy and personal freedoms, resistance to such measures may be more prevalent.^38^ In other words, in countries where individualism is dominant, compliance with institutional quarantine and isolation may be poor,^39^ while the compliance rate in settings where communalism subsists may be high. Balancing the need for public health with respect for individual liberties becomes a delicate cultural negotiation.

Even the concept of “isolation center”, which was widely used in different countries such as Nigeria, Ghana, India, Kenya and others during disease outbreaks to denote quarantine facilities, created negative notions in the communities. Staying in Q&I facilities was reported to have engendered stigmatization and discrimination against people who were admitted.^40^, ^41^ Consequently, fear and misunderstanding led people to avoid quarantine.^42^, ^43^ The impact of Q&I on familial and community structures is another cultural consideration. In African cultures, where extended family networks are prevalent, the separation of family members during quarantine can have profound emotional and practical implications. Cultural rituals and practices around caregiving may clash with isolation measures, posing challenges for individuals accustomed to close familial support during times of sickness. Invariably, social capital affects responses to public health emergency measures.^44^

Religious beliefs also influence responses to quarantine and isolation. Some communities may find solace and meaning in religious practices during challenging times, while others may grapple with the limitations placed on communal worship and religious gatherings.^45^, ^46^ Understanding and respecting diverse religious practices is essential to striking a balance between public health requirements and religious freedom. Likewise, changes in cultural norms and practices associated with such major life events as the care of the dead also affected compliance with institutional quarantine and isolation. Burial practices are dictated by religious beliefs; however, during infection outbreaks, religious burial traditions like cleansing or bathing and close contact with dead bodies, as being practiced by Muslims and Christians,^47^ were discouraged by authorities managing infectious disease outbreaks. In Italy and India, preventing families from burying their dead as culturally appropriate during disease outbreaks had adverse effects on their mental health and the grieving period.^48^ This rationalizes the negative attitude towards medically enforced burial practices, which deny families access to their dead.^49^

Stigmatization is a pervasive cultural issue associated with quarantine and isolation.^40^, ^50^ Certain cultures may stigmatize individuals who have been isolated or are perceived as being at a higher risk of infection. This stigma can lead to discrimination, social exclusion, and mental health challenges for those affected.^51^ Addressing cultural beliefs and dispelling misinformation becomes crucial in mitigating the negative consequences of stigma during public health crises.^52^, ^53^ Moreso, socioeconomic factors intersect with cultural issues, as marginalized communities often bear a disproportionate burden during quarantine and isolation.^53^ Economic disparities, a lack of access to healthcare, and limited resources can exacerbate the challenges faced by culturally diverse populations, highlighting the importance of equitable public health interventions.^54^

### 4.1 Limitations

This scoping review aims to synthesize evidence from multiple studies to draw comprehensive and reliable conclusions. Conversely, with only one study, the results are more specific to the context, population, and methodology of the reviewed study. This could affect the ability to generalize the findings to a broader context. Impliedly, the results are derived from limited evidence and could be tempered as such. The initial aim was to provide robust evidence by combining data from several studies to enhance the empirical utility and potential policy relevance of the results. With only one eligible article, the evidence is not adequately robust though better than empty reviews (without any eligible article) which are also significant.^55^, ^56^ However, the dearth of research concerning cultural issues concerning Q&I indicates gaps in the literature that need to be addressed through additional studies. While this review lacks the diversity of evidence, which hinders a thorough exploration of the topic, the study is complemented by a more nuanced discussions which highlight more cultural issues beyond the eligible article. Hence, the conclusion is thoughtful and the recommendations are reasonably actionable. However, future research should aim to expand the evidence base, using diverse methodologies and theoretical perspectives to support more robust conclusions.

## 5 CONCLUSION

The execution of Q&I should be evaluated in light of local cultural factors rather than just in terms of health technology, emergency measures and care standards. Emergency healthcare needs to always respect diverse cultural practices in a way that strikes a balance between public health requirements and such cultural practices. The cultural issues surrounding Q&I are complex and multifaceted. Recognising and addressing these issues requires a nuanced understanding of diverse cultural contexts. Culturally sensitive public health strategies, clear communication, and collaborative efforts with communities can help build trust, promote adherence to guidelines, and minimise the negative social and psychological impacts of Q&I measures.

Communication strategies also play a pivotal role in navigating cultural sensitivities during Q&I. Culturally competent messaging that respects diverse belief systems, languages, and communication styles is crucial to fostering trust and encouraging adherence to public health guidelines. Ultimately, navigating cultural considerations during public health crises is essential for fostering resilience and social approval within societies facing the challenges of contagious diseases. Hence, future studies should examine other cultural issues (such as social inequality, social capital, diversity and grieving practices, among others) beyond the funeral rites in Africa.

## AUTHOR CONTRIBUTIONS

**Ebunoluwa Oduwole**: Conceptualization; Writing—review and editing; Project administration; Resources; Supervision; Funding acquisition. **Jimoh Amzat**: Conceptualization; Investigation; Funding acquisition; Writing—original draft; Methodology; Validation; Visualization; Writing—review and editing; Software; Formal analysis; Project administration; Data curation; Supervision; Resources. **Olusola Aluko‐Arowolo**: Writing—review and editing; Resources; Conceptualization; Funding acquisition. **Rotimi Afolabi**: Conceptualization; Writing—review and editing; Resources; Funding acquisition. **Isaac Akinkunmi Adedeji**: Conceptualization; Writing—review and editing; Resources; Funding acquisition. **Saheed Akinmayowa Lawal**: Conceptualization; Writing—review and editing; Resources; Funding acquisition. **Ige Angela Temisan**: Conceptualization; Writing—review and editing; Resources; Funding acquisition. **Ayoyinka Oludiran**: Conceptualization; Writing—review and editing; Resources; Funding acquisition. **Kafayat Aminu**: Resources; Writing—review and editing. **Afeez Abolarinwa Salami**: Methodology; Investigation; Writing—original draft; Resources; Software; Visualization; Validation. **Kehinde Kazeem Kanmodi**: Investigation; Writing—original draft; Methodology; Validation; Visualization; Writing—review and editing; Software; Formal analysis; Project administration; Data curation; Resources; Supervision; Funding acquisition; Conceptualization.

## CONFLICT OF INTEREST STATEMENT

Kehinde Kazeem Kanmodi is an Editorial Board member of *Health Science Reports* and a coauthor of this article. To minimize bias, they were excluded from all editorial decision‐making related to the acceptance of this article for publication. Other authors have no conflict of interest to declare.

## ETHICAL STATEMENT

Not applicable. This study did not collect data from human or animal subjects but from an open research repository.

## TRANSPARENCY STATEMENT

The lead author Kehinde Kazeem Kanmodi affirms that this manuscript is an honest, accurate, and transparent account of the study being reported; that no important aspects of the study have been omitted; and that any discrepancies from the study as planned (and, if relevant, registered) have been explained.

## Supporting information

Supporting information.

## Data Availability

Data sharing is not applicable to this article as no new data were created or analyzed in this study. Data sharing does not apply to this article as no new data were created or analyzed in this study. All authors have read and approved the final version of the manuscript. The manuscript guarantor, Kehinde Kazeem Kanmodi, had full access to all of the data in this study and takes complete responsibility for the integrity of the data and the accuracy of the data analysis.
